# Do Olive and Fish Oils of the Mediterranean Diet Have a Role in Triple Negative Breast Cancer Prevention and Therapy? An Exploration of Evidence in Cells and Animal Models

**DOI:** 10.3389/fnut.2020.571455

**Published:** 2020-10-06

**Authors:** Micah G. Donovan, Ornella I. Selmin, Barbara J. Stillwater, Leigh A. Neumayer, Donato F. Romagnolo

**Affiliations:** ^1^Interdisciplinary Cancer Biology Graduate Program, The University of Arizona, Tucson, AZ, United States; ^2^University of Arizona Cancer Center, The University of Arizona, Tucson, AZ, United States; ^3^Department of Nutritional Sciences, The University of Arizona, Tucson, AZ, United States; ^4^Department of Surgery, Breast Surgery Oncology, The University of Arizona, Tucson, AZ, United States

**Keywords:** mediterranean diet, omega 3 fatty acids, mono saturated fatty acid, triple negative breat cancer, olive and fish oil

## Abstract

Breast cancer is the most common malignancy and cause of cancer-related mortality among women worldwide. Triple negative breast cancers (TNBC) are the most aggressive and lethal of the breast cancer molecular subtypes, due in part to a poor understanding of TNBC etiology and lack of targeted therapeutics. Despite advances in the clinical management of TNBC, optimal treatment regimens remain elusive. Thus, identifying interventional approaches that suppress the initiation and progression of TNBC, while minimizing side effects, would be of great interest. Studies have documented an inverse relationship between the incidence of hormone receptor negative breast cancer and adherence to a Mediterranean Diet, particularly higher consumption of fish and olive oil. Here, we performed a review of studies over the last 5 years investigating the effects of fish oil, olive oil and their components in model systems of TNBC. We included studies that focused on the fish oil ω-3 essential fatty acids docosahexaenoic acid (DHA) and eicosapentaenoic acid (EPA) in addition to olive oil polyphenolic compounds and oleic acid. Both beneficial and deleterious effects on TNBC model systems are reviewed and we highlight how multiple components of these Mediterranean Diet oils target signaling pathways known to be aberrant in TNBC including PI3K/Akt/mTOR, NF-κB/COX2 and Wnt/β-catenin.

## Introduction

Breast cancer is the most common malignancy and cause of cancer-related mortality among women worldwide (1). Triple negative breast cancer (TNBC) refers to a heterogeneous subclassification of breast tumors that by definition lack expression of the estrogen receptor (ER)α, progesterone receptor (PR) and human epidermal growth factor receptor (HER)2. These tumors account for ~15% of breast cancer cases and are more likely to occur in women that are young, obese (2), from African-American or Hispanic descent (3), or harbor germline *BRCA1* mutations (4). Triple negative breast cancers carry a worse prognosis than hormone receptor-positive (luminal A and luminal B) and HER2-enriched (HER2+) breast tumors (5). For TNBC, the 5y survival rate for stage I patients is ≥85% compared with ≥99 and ≥94% for luminal (A and B) and HER2+ breast cancer, respectively. Whereas, in the metastatic setting, the median survival for TNBC is ~10–13 mo compared to ~5 y for non-TNBC.

Poor prognosis of TNBC is due in part to an incomplete understanding of its molecular etiology and thus reliance on non-targeted chemotherapy for treatment. Moreover, compared with the other molecular subtypes of breast cancer, TNBC have an increased tendency for visceral and central nervous system metastases (6, 7) and carry a higher risk of relapse and distant recurrence (8, 9). Optimal chemotherapy regimens for TNBC remain elusive, although some benefit has been observed in clinical trials with neoadjuvant platinum-based therapeutics and adjuvant use of paclitaxel (2). Poly (ADP-ribose) polymerase (PARP) inhibitors have also shown promise against metastatic *BRCA-*mutated HER2-negative breast tumors (10). Despite these advances, the development of interventional approaches that suppress the initiation and progression of TNBC and have minimal side effects would be of great benefit.

Diet is implicated as a non-genetic risk effector in breast cancer etiology that can have both beneficial and deleterious influences (11). Moreover, cancer prevention by dietary bioactive components is of great interest as these compounds are cost effective and safe in addition to having anti-tumorigenic properties (12). The Mediterranean Diet (MD) describes the dietary patterns of cultures that have surrounded the Mediterranean sea for millennia (13). There is no absolute definition of what constitutes a MD, however it is generally accepted to be a diet that (1) is rich in plant-based foods (i.e., vegetables, fruit, whole grains, legumes, etc.); (2) uses olive oil as the principle fat source; (3) has low intakes of red and processed meat, saturated fat, and refined sugars; (4) advocates low-to-moderate intake of low-fat dairy and moderate consumption of fish; and (5) emphasizes moderate intake of alcohol (mostly red wine) with meals (14). Several scoring systems have been derived in order to quantitate MD adherence in the context of studying health outcomes (15–17) (Table 1). Adherence to the MD has been demonstrated to be inversely associated with multiple health maladies including cardiovascular disease (18), diabetes (19), Alzheimer's disease (20), and cancer (21).

**Table 1:** Scoring systems used to quantitate MD adherence, derived from (15–17).

**Scoring system**	**Components**	**Scoring**
Mediterranean Diet Score (MDS)	Alcohol, cereals, dairy*, fish, fruits and nuts, legumes, meat and meat products*, MUFA:SFA ratio, vegetables	Range 0–9 One point for intake ≥ study-specific median of components without* One point for intake < study-specific median of components with* One point for alcohol intake of 5–25 g/day for women and 10–50 g/day for men (otherwise 0)
Alternate Mediterranean Diet Score (aMED)	Alcohol, fish, fruit, legumes, MUFA:SFA, nuts, red and processed meat*, vegetables, whole grains	Range: 0–9 One point for intake ≥ study-specific median of components without* One point for intake < study-specific median of components with* One point for alcohol intake of 5–15 g/d
Adapted Relative Mediterranean Diet Score (arMED)	fruits, vegetables, legumes, fish, olive oil, cereals, dairy*, meat products*	Range: 0–16 0–2 points assigned to country-specific tertiles of intake for components without* Scoring inverted for components with*

*MUFA, monounsaturated fatty acids; SFA, saturated fatty acids*.

With respect to breast cancer, both clinical trials (22) and epidemiological studies (23, 24) have suggested a protective influence of MD adherence (Table 2). The PREDIMED study was a multicenter randomized primary prevention trial comparing the effect of following a MD against a low-fat diet (control) on breast cancer incidence in women (*n* = 4,282, 60–80 y) at high cardiovascular disease risk (22). In fully adjusted models, a group adhering to a MD rich in extra virgin olive oil (EVOO) had significantly less overall breast cancer cases compared with the control group (HR = 0.32, 95%CI: 0.13–0.79). Meta-analyses investigating the association between MD adherence and BC incidence have reported either modest changes in risk ratio (RR = 0.93–0.86) (23, 24) or no significant associations (25, 26). However, of the latter studies, one (26) found significant risk reductions for ER- tumors (RR = 0.84) upon subgroup stratification, whereas no associations were found for total breast cancer or ER+ breast cancer.

**Table 2:** Effect of MD adherence on BC incidence reported from studies in human populations.

**Study design**	**Intervention/variable**	**Outcome**	**Effect/association**	**HR/OR/RR (95% CI)**	**References**
**Clinical trials**					
	MD supplemented with EVOO	BC incidence	**Decreased**	**HR = 0.32 (0.13–0.79)**	(22)
**Meta-analyses**					
	MD adherence	BC incidence	**Inverse**	**RR = 0.93 (0.87−0.99)**	(23)
	MD adherence	BC incidence	**Inverse**	**RR = 0.92 (0.87–0.96)**	(24)
	MD adherence	BC incidence	N/A	RR = 0.96 (0.90–1.03)	(25)
	MD adherence	BC incidence ER+ BC incidence ER- BC incidence	N/A N/A **Inverse**	RR= 0.94 (0.87–1.01) RR= 0.92 (0.85–1.01) **RR= 0.84 (0.73–0.97)**	(26)
**Observational**					
	aMED adherence	BC incidence ER+ BC incidence PR+ BC incidence ER/PR+ BC incidence ER- BC incidence PR- BC incidence ER/PR- BC incidence	N/A N/A N/A N/A **Inverse** **Inverse** **Inverse**	HR = 0.87 (0.72–1.06) HR = 0.87 (0.69–1.10) HR = 0.90 (0.69–1.19) HR = 0.91 (0.69–1.21) **HR = 0.60 (0.39–0.93)^a^** **HR = 0.72 (0.52–1.05)^a^** **HR = 0.61 (0.36–1.01)^a^**	(26)
	arMED adherence	BC incidence ER/PR+ BC incidence ER/PR- BC incidence P.M. BC incidence P.M. ER/PR+ BC incidence P.M. ER/PR- BC incidence Pr.M BC incidence Pr.M. ER/PR+ BC incidence Pr.M. ER/PR- BC incidence	**Inverse** N/A N/A **Inverse** N/A **Inverse** N/A N/A N/A	**HR = 0.94 (0.88, 1.00)^a^** HR = 0.92 (0.85, 1.00) HR = 0.84 (0.69, 1.02) **HR = 0.93 (0.87, 0.99)^a^** HR = 0.92 (0.85, 1.01) **HR = 0.80 (0.65, 0.99)^a^** HR = 0.97 (0.81, 1.15) HR = 0.86 (0.66, 1.13) HR = 1.09 (0.65, 1.82)	(17)
	aMED adherence “*a posteriori”* MD^b^ adherence	ER/PR+, HER2- BC incidence HER2+ BC incidence TNBC BC incidence ER/PR+, HER2- BC incidence HER2+ BC incidence TNBC BC incidence	N/A N/A **Inverse** **Inverse** N/A **Inverse**	OR = 0.91 (0.82–1.01) OR = 0.94 (0.80–1.11) **OR = 0.80 (0.65–0.99)** **OR = 0.83 (0.73–0.94)** OR = 0.86 (0.71–1.04) **OR = 0.63 (0.50–0.78)**	(27)
	*“a posteriori”* MD^b^ adherence	ER/PR+ HER2+ TNBC incidence	N/A N/A N/A	OR = 0.91 (0.67–1.23) OR = 0.86 (0.51–1.47) OR = 0.73 (0.34–1.55)	(28)
	aMED adherence	BC incidence ER/PR+ BC incidence ER/PR- BC incidence TNBC incidence	**Inverse** N/A N/A N/A	**HR = 0.89 (0.76–1.05)^a^** HR = 0.87 (0.72–1.07) HR = 0.80 (0.50–1.29) HR = 0.66 (0.37–1.19)	(29)

a *Significant P-trend reported in bold*.

b *Characterized by high intakes of fish, vegetables, legumes, boiled potatoes, fruits, olives and vegetable oil, and a low intake of juices*.

*aMED, alternate Mediterranean Diet Score; arMED, adapted relative Mediterranean diet*.

To date, no meta-analysis has investigated the association between MD adherence and TNBC incidence specifically. However, individual observational studies (i.e., not meta-analyses) suggest a potential inverse relationship (Table 2). A cohort study using data from the Netherlands Cancer Registry and the Dutch Pathology Registry reported an inverse association between higher adherence to the alternate Mediterranean Diet Score (aMED) and incidence of ER- breast cancer. Less significant inverse associations were also found for PR- and ER-/PR- tumors. However, no associations were found for ER+, PR+ or ER+/PR+ tumors (26). In a subgroup analysis of data from the European Prospective Investigation into Cancer and Nutrition cohort (EPIC) study, authors found adherence to the adapted relative Mediterranean diet (arMED) index was associated with decreased odds of ER-/PR- tumors in post-menopausal women (17). A case-control study using cases from the Spanish GEICAM Breast Cancer Research Group reported lower odds of TNBC incidence with adherence to both an “*a priori”* and “*a posteriori*” MD pattern (27). A more recent case-control study in the Spanish population did not find a statistical significance (*P*-trend = 0.322) in the lower odds ratio calculated for TNBC incidence, however authors suggested this was likely due to a small number of cases (*n* = 76) (28). A study investigating the relationship in population of U.S. women with a family history of breast cancer (*n* = 33) suggested higher MD adherence trended toward a risk reduction of 34%, although the trend was not statistically significant (*p* = 0.07), perhaps due to small sample size (29).

With respect to mortality, one study has reported an inverse relationship between following a MD and non-BC related death (RR = 0.39), although no association was found for breast cancer-specific mortality (30). To our knowledge, no studies have investigated specifically the effect of MD adherence on TNBC mortality. However, given the observation that the MD has a more distinct relationship with hormone receptor-negative breast tumor incidence, the capacity to detect an effect of MD adherence on TNBC outcomes may be lost in non-stratified analyses of breast cancer cases. Results from the PREDIMED trial and subgroup analyses in epidemiological studies implicate the protective effect of the MD against breast cancer incidence is attributable in part to higher intakes of olive oil and fish/fish oil (31–33). Moreover, preclinical studies have documented protective effects of both olive (34–36) and fish oil (37–39) in rodent mammary tumor models. Given the inverse association between higher consumption of fish and olive oil and breast cancer incidence, the purpose of this article was to review studies that investigated the effects of olive oil, fish oil and their components on model systems of TNBC.

## Methods

**Figure 1:**
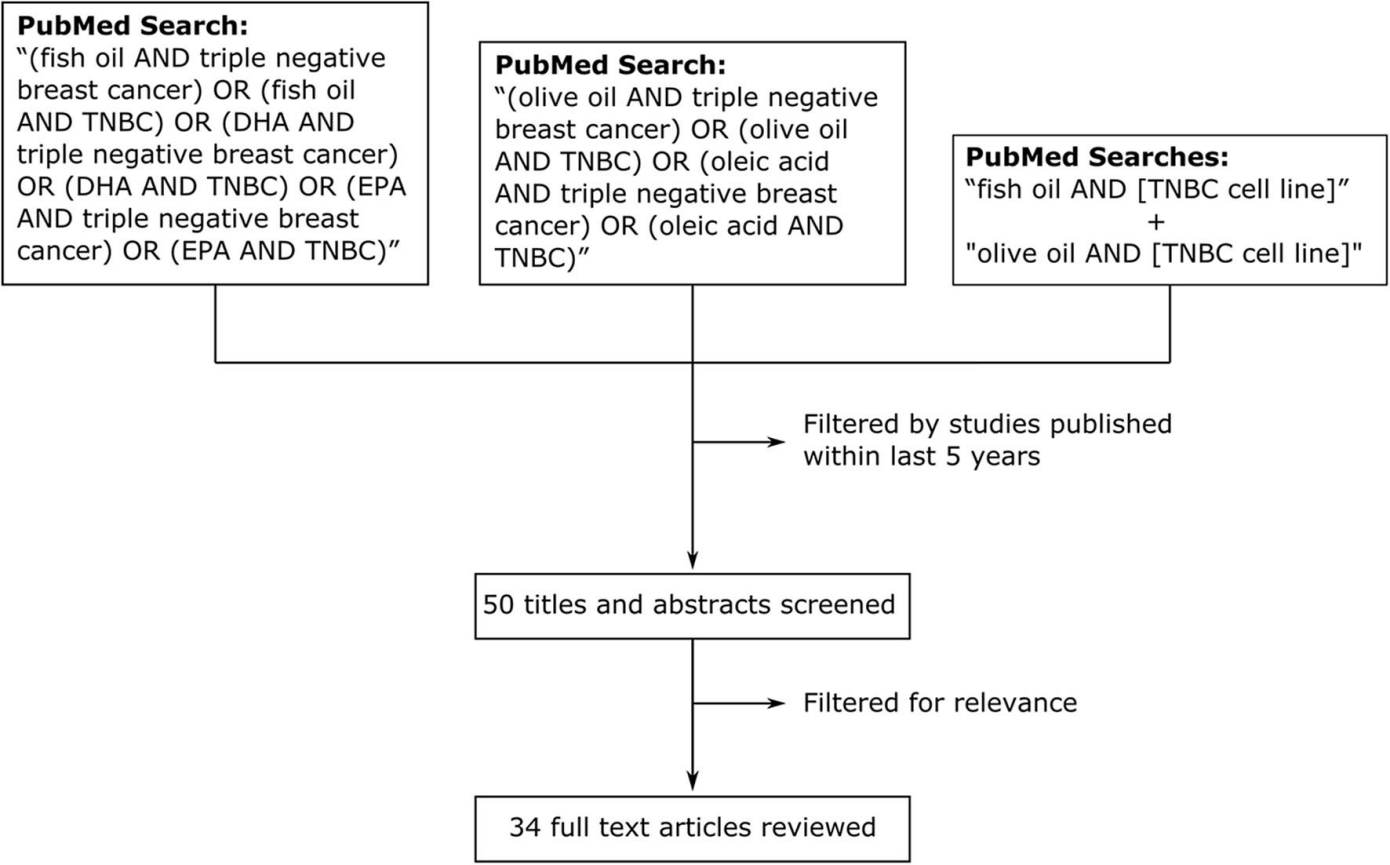
Methodology for review of effects of fish oil, olive oil, and their components on model systems of TNBC in studies published within the last 5 y. We searched PubMed with the search queries “(fish oil AND triple negative breast cancer) OR (fish oil AND TNBC) OR (DHA AND triple negative breast cancer) OR (DHA AND TNBC) OR (EPA AND triple negative breast cancer) OR (EPA AND TNBC)” and “(olive oil AND triple negative breast cancer) OR (olive oil AND TNBC) OR (oleic acid AND triple negative breast cancer) OR (oleic acid AND TNBC).” We also searched with the terms “fish oil” and “olive oil” in combination with the names of the 27 most commonly cited TNBC cell lines (separate searches).

We performed a traditional review of studies over the last 5 years (Figure 1) investigating the effects of fish oil, olive oil and their components in model systems of TNBC. Searches of the PubMed database with the queries “(fish oil AND triple negative breast cancer) OR (fish oil AND TNBC) OR (DHA AND triple negative breast cancer) OR (DHA AND TNBC) OR (EPA AND triple negative breast cancer) OR (EPA AND TNBC)” and “(olive oil AND triple negative breast cancer) OR (olive oil AND TNBC) OR (oleic acid AND triple negative breast cancer) OR (oleic acid AND TNBC)” returned a total of 17 and 11 articles, respectively, published over the last 5 y. As a supplement, and to ensure thorough examination of the topic, we performed additional searches with the terms “fish oil” and “olive oil” in combination with the names of the 27 most commonly cited TNBC cell lines (40). All searches returned a combined total of 50 studies published within our selected time frame (5 y). After filtering for relevance, we identified a total of 34 articles reviewed below (41–74). Some studies that were published outside our selected time frame (>5 y) were identified in *post-hoc* searches and also reviewed to enhance the perspective and provide additional commentary. Searches for fish oil returned studies that focused on the ω-3 essential fatty acids docosahexaenoic acid (DHA) and eicosapentaenoic acid (EPA). For olive oil, we included studies looked at several of the polyphenolic compounds that are abundant in olive oil and the 18:1 monounsaturated fatty acid oleic acid, which is the predominant free fatty acid in olive oil (75). The studies we reviewed performed cell culture experiments with TNBC cell lines and some of these (41, 48, 53–56, 69, 71, 72) extended their experiments *in vivo*.

## Fish Oil and TNBC: Review

### Effects on Proliferation and Growth

**Figure 2:**
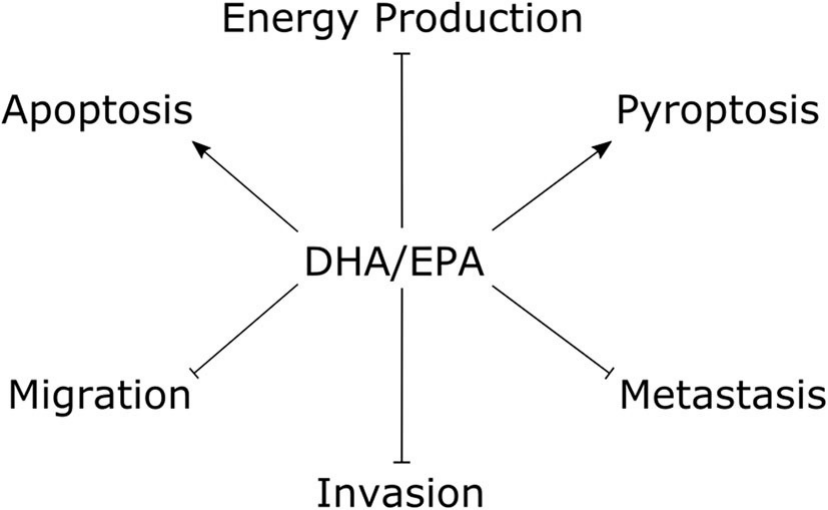
The tumor suppressive effects of fish oil ω-3 essential fatty acids in TNBC cells. Studies performed within the last 5 y documented treatment with DHA/EPA have anti-proliferative effects on TNBC cells. Mechanistic studies with DHA showed pro-apoptotic and pro-pyroptotic effects, with inhibitory effects on glycolytic and mitochondrial energy metabolism. DHA and EPA showed suppressive effects on TNBC cell migration and invasion, and DHA decreased experimental metastasis. Arrows indicate stimulus, blunted lines indicate repressive effects. DHA, docosahexaenoic acid; EPA, eicosapentaenoic acid.

The tumor suppressive effects of fish oil ω-3 essential fatty acids on TNBC cells are summarized in Figure 2. Several studies documented anti-proliferative effects of both DHA and EPA in preclinical models of TNBC (41–46). Some suggest the effect of DHA may be stronger than that of EPA (41, 44). For example, in MDA-MB-231 TNBC cells, DHA treatment for 24 h decreased cell viability at a range of concentrations starting at 5 μM (20% growth inhibition) up to 50 μM (85% growth inhibition) (41). On the other hand, EPA did not produce a significant reduction until 25 μM (~15% growth inhibition) and only 60% growth inhibition was observed at the highest dose (50 μM). Both compounds had a slight anti-proliferative effect in T47D luminal breast cancer cells, however the maximal effect was only ~40% growth inhibition by DHA at 50 μM. Another study reported the 24 h half maximal inhibitory concentration (IC_50_) for cell viability was ~130 μM for DHA and ~160 μM for EPA in MDA-MB-231 cells, and ~120 μM for DHA and ~180 μM for EPA in MCF7 luminal breast cancer cells (44). On the other hand, EPA and DHA seemed to have similar anti-proliferative efficacy in a study that documented an ~25% decrease in the number of MCF7 (luminal A), SK-BR-3 (HER2+), MDA-MB-231 (TNBC) and HCC1806 (TNBC) cells treated with either EPA or DHA (80 μM) for 72 h (42). Notably, 160 μM of either compound decreased MDA-MB-231 cell count by ~75% and approached 100% cytotoxic efficiency against the HCC1806 cell line.

Treatment of MDA-MB-231 cells with DHA (10–25 μM) lead to an accumulation of the subG1 cell population with concomitant increases in PARP cleavage and caspase-3 activity (41). Moreover, flow cytometry analysis of Annexin V and propidium iodide (PI) staining indicated DHA promoted apoptotic cell death in both MDA-MB-231 and HCC1806 TNBC cells (42). Interestingly, one study found co-treatment with vitamin D3 enhanced the pro-apoptotic effect of both EPA and DHA in MCF7, SK-BR-3, and MDA-MB-231 cells (46).

In addition to apoptosis, one study (43) showed DHA may also trigger TNBC cell death through induction of pyroptosis, a cell death program distinct from apoptosis that is triggered by inflammatory signaling and mediated by inflammasomes (76). Here, DHA treatment had a cytotoxic effect on MDA-MB-231 cells with a half maximal effective concentration (EC_50_) of 101.6 μM (43). Intriguingly, DHA treatment had no effect on the viability of non-malignant MCF10A mammary cells or peripheral blood mononuclear cells. Pyroptosis is initiated by activation of Caspase-1, which proteolytically cleaves interleukin (IL)-1β and gasdermin D (GSDMD) to their active forms (77, 78). The latter molecule, GSDMD, forms membrane pores, which ultimately triggers pyroptotic cell death. Indeed, MDA-MB-231 cells treated with DHA had higher levels of activated Caspase-1 and GSDMD, enhanced IL-1β secretion, and other markers of pyroptotic cell death (43).

Changes in TNBC cell proliferation and growth resultant from DHA treatment may also be related to modulation of cell metabolism and bioenergetics. Treatment with DHA dose-dependently decreased both glycolytic and mitochondrial metabolism in both BT-474 and MDA-MB-231 TNBC cells, however no effect was observed in the non-malignant MCF10A cell line (47). These specific effects in the TNBC cells were linked to DHA-mediated decreases in hypoxia-inducible factor 1 (HIF1)α, a key regulator of glycolytic metabolism and the Warburg effect in cancer cells (79). Moreover, in TNBC cells, DHA decreased HIF1α transcriptional activity and expression of its targets glucose transporter 1 (GLUT 1) and lactate dehydrogenase (LDH), which are key mediators of glycolytic metabolism in cancer cells (47).

A greater anti-proliferative effect on MDA-MB-231 cells was observed with the endocannabinoid derivatives of DHA and EPA, docosahexaenoyl-ethanolamine (DHEA) and eicosapentaenoyl-ethanolamine (EPEA), compared to the parent compounds (44). In these experiments the IC_50_ for cell viability was ~130 μM for DHA after both 24 and 48 h; ~80 μM and ~130 μM for DHEA at 24 and 48 h, respectively; ~160 μM for EPA after 24 and 48 h; and ~80 μM and ~50 μM for EPEA at 24 and 48 h respectively. Changes in proliferation induced by the ω-3 compounds were shown to be regulated by the endocannabinoid receptors CB1 and CB2. Co-treating cells with an antagonist specific to CB1 decreased the anti-proliferative effect of DHA and EPEA and completely ablated the effects of DHEA, but increased EPA efficacy. On the other hand, a CB2-specific antagonist increased the efficacy of DHA and DHEA but decreased the effect of EPA and EPEA. The effect of DHA and DHEA was also increased by antagonizing both CB1 and CB2 in co-treatment experiments, whereas dual antagonism attenuated the efficacy of EPA and EPEA. Of note, this study also documented that none of the compounds tested had an effect on viability of the non-malignant MCF10A cell line.

There may be cell-specific and metabolite-specific effects of DHA on BC cell proliferation. For example, treatment with DHA (100 μM for 96 h) significantly decreased MDA-MB-231 TNBC cell proliferation by ~85% compared to controls, whereas no statistically significant effect was observed in BT-549 TNBC cells (45). On the other hand, 4-OXO-DHA, an oxidized DHA metabolite, had a more potent anti-proliferative effect in MDA-MB-231 cells and decreased BT-549 proliferation by ~85% compared to control cells. In luminal breast cancer cells, DHA and 4-OXO-DHA increased MCF7 cell proliferation, whereas both metabolites decreased proliferation of T47D and SK-BR-3 cells. Interestingly, this study suggests different BC subtypes, and even different tumors of the same subtype, may have differential responses to DHA. However, other studies showed DHA and EPA had similar IC_50_ values against cell viability in both MCF7 and MDA-MB-231 cells (44).

Gene expression analyses have been used to stratify TNBC into multiple subtypes. For example, Lehmann et al. have proposed four TNBC tumor specific subtypes referred to as basal like (BL)1, BL2, mesenchymal and luminal androgen receptor (LAR) (80). Basal-like tumors that are triple negative and have low expression of luminal differentiation markers, high expression of genes involved with the epithelial-to-mesenchymal transition (EMT) and immune response, and are enriched with cancer stem cells are referred to as claudin-low (CL) TNBC (81). One study demonstrated supplementation with EPA+DHA (0.025%) significantly decreased growth of models of both claudin low (CL) and basal-like (BL) murine mammary tumors in mice fed an obesogenic diet (60% fat) (48). Mice were orthotopically injected with BL or CL cells derived from murine MMTV-Wnt-1 tumors. Compared with mice fed only the obesogenic diet, mice that received the EPA+DHA supplement had significantly less tumor area and weight. Analysis of tumors showed the EPA+DHA supplement increased the level of cleaved caspase-3 compared to controls and attenuated upregulation of inflammation markers such as cyclooxygenase-2 (COX2) and phospho-p65, the major subunit for NF-kB (48).

In Fat-1 mice, the tumor volume orthotopic EO771 cell xenografts was decreased by ~80%, which was associated with decreased NF-κB binding affinity, compared to wildtype (WT) controls (41). Fat-1 carry a transgene for *Caenorhabditis elegans* ω3 desaturase (*fat-1* gene), which causes them to produce high amounts of endogenous ω-3 polyunsaturated fatty acids (PUFA) from endogenous ω-6 PUFA. The effect of DHA on tumor NF-kB activity was corroborated by cell culture experiments using MDA-MB-231 cells (41). Treatment of MDA-MB-231 cells with DHA (0–50 μM) dose-dependently decreased cell viability, which was associated with an accumulation of the subG1 cell population and concomitant increases in PARP cleavage and caspase-3 activity. These effects were attributed in part to DHA-induced decreases in the expression of COX2 and VEGF and promoter activity of NF-kB.

### Effects on Migration, Invasion and Metastasis

Compared with the other BC subtypes, TNBC have an increased propensity for visceral and central nervous system metastases (6, 7). The epithelial-to-mesenchymal transition (EMT) is a process whereby epithelial cells lose polarity and cell-cell contacts and increase migratory capacities, which facilitates movement into surrounding tissue (invasion) and seeding in distant locations (82). Several studies have documented suppressive effects of fish oil ω-3 fatty acids on EMT characteristics including cell migration and invasion, which are key to the metastatic process. Transforming growth factor (TGF)β is a cytokine that promotes EMT of epithelial cells (82). In two different TNBC cell lines, MDA-MB-435 and Hs578T, DHA (25 μM) suppressed transforming growth factor (TGF)β-mediated increases in the expression of the EMT mediators gremlin (GREM)1, N-cadherin, vimentin, and Slug (49). Stimulation of MDA-MB-435 cells with TGFβ had a pro-migratory effect in wound healing assays that was attenuated by either GREM1 silencing or treatment with DHA. Authors attributed the effect of DHA in part to inhibition of ERK phosphorylation.

Treatment with DHA (100 μM for 24 h) also decreased invasion of MDA-MB-231 cells in Boyden chamber assays by 60% compared to control cells (50). These effects were abrogated upon co-treatment with siRNA against keratin, type II cytoskeletal 1 (KRT1), which was significantly upregulated upon treatment with DHA alone. Downregulation of KRT1 has been shown to occur as human mammary epithelial cells undergo malignant transformation mediated by retroviral addition of Simian Virus 40 Early Region (*SV40 ER*), exogenous human telomerase reverse transcriptase (*hTERT*), and an activated, oncogenic form of hRAS (*hRAS-V12*) (83).

The EMT has been shown to be triggered by a variety of factors including Wnt/β-catenin (84), fascin-1 (85), and STAT3 (86) among others. The interaction of WNT ligands with their receptors leads to stabilization and nuclear translocation of β-catenin, a transcription factor regulating genes involved in proliferation, differentiation/development, and migration (87). Silencing fascin-1 suppresses EMT in part through preventing nuclear localization of β-catenin (85). On the other hand, STAT3 promotes EMT through upregulation of Twist-1 (86). The Hs578T cell line is a highly migratory model of TNBC that expresses high levels of fascin-1, β-catenin and STAT3α (51). Treatment of Hs578T cells with DHA (100 μM for 24 h) significantly decreased their migration in wound healing assays, which was linked to a dose-dependent decrease in fascin-1, β-catenin and STAT3α expression. Pretreatment with DHA was also shown to suppress 12-O-tetradecanoylphorbol-13- acetate (TPA)-induced increases in MCF7 cell migration, Wnt-1 protein levels and secretion, and β-catenin expression.

Hypoxia in the tumor microenvironment is associated with an increased risk for metastasis and mortality (88). The cellular response to hypoxia is largely regulated by the activity of the HIF1 and HIF2 transcription factors. Targets of the HIF transcription factors include genes involved in several aspects of the EMT and metastasis including matrix metalloproteinase (MMP)2, MMP9, vimentin, and snail 1 and 2 among many others (89). Matrix metalloproteinases facilitate invasion by promoting the degradation of the extracellular matrix and basement membrane (88). On the other hand, vimentin is an intermediate filament protein that promotes cell migration through regulation of microtubules and actomyosin networks (90). Vimentin expression is considered an EMT marker and is associated with poor prognosis in multiple cancer types (91). Under both normoxic and hypoxic conditions, DHA (100 μM) decreased migration of MDA-MB-231 cells in wound healing assays (52). This was shown to be associated with increased miR-194 expression and decreased miR-106b, which led to downregulation of vimentin, matrix metalloproteinase (MMP)2, and MMP9 and increased talin2. Talins are cytoskeletal proteins that link integrin adhesion proteins with F-actin in cells (92) and depletion of talin2 in MDA-MB-231 cells decreased their migration, invasion and expression of HIF-1α, in part through downregulation of AKT/mTOR signaling (93). Interestingly, previous studies showed DHA treatment inhibited growth of MDA-MB-453 TNBC cells in part through inhibition of Akt phosphorylation (94), which may underlie its effect on talin2 expression in MDA-MB-231 cells (52).

Integrins are transmembrane heterodimer proteins that are formed through combinations of α and β subunits (95). Integrins act as signaling molecules and transducers of mechanical stimuli and have significant roles in cell migration and invasion. Targeting β -containing integrins (e.g., α_v_β_3_) has shown promise in preclinical studies to reverse EMT and reduce migration and invasion of MDA-MB-231 and BT549 TNBC cells (96). Brown et al. used transwell assays in both the absence and presence of a synthetic basement membrane matrix to assess changes in MDA-MB-231 cell migration and invasion, respectively (44). Compared to controls, only DHA lowered migratory capacity (~70%), however both DHA and EPA decreased invasion by ~70 and ~55%, respectively. Western blotting showed DHA treatment of MDA-MB-231 cells decreased protein levels of the β3 integrin subunit, matrix MMP1 and VEGF, whereas EPA only affected levels of β3 integrin (44). It has been shown that metastatic TNBC cells have higher expression of MMP1, MMP9, MMP13, and vascular endothelial growth factor (VEGF) among several other factors involved in EMT (97). While the function of MMP proteins is to degrade the extracellular matrix, VEGF is a permeability factor that promotes angiogenesis and cancer cell intravasation (89). Activity of VEGF is also needed for lymphatic migration of tumor cells (97).

Sites of TNBC metastatic seeding include the viscera and central nervous system rather than the bone, which is common in ER-positive breast cancer. Gene expression analyses of cells from BC brain metastases demonstrated COX2 as a key facilitator of cancer cell entry passed the blood-brain-barrier (98). Moreover, high tumor expression of COX2 is associated with worse 5 y survival in TNBC patients (99). A study in the MDA-MB-231 cell line documented 10 μM treatment with DHA produced an ~10-fold decrease in invasion through Matrigel chambers, which was associated with downregulation of MMP2 and MMP9 expression and COX2 promoter activity (41). Treatment with DHA was also shown to attenuate the dose-dependent induction of MMP2 and MMP9 mediated by either prostaglandin E_2_ or arachidonic acid. Authors also demonstrated NF-κB signaling was suppressed by DHA treatment. Higher activity of NF-κB has been observed in TNBC cells (100) and has been shown to promote invasion and metastasis (101). Given the effect of DHA on MMP2, MMP9, COX2, and NF-κB, authors studied its impact on metastasis by injecting EO771 murine BC cells into the tail veins of wildtype (WT) and Fat-1 mice (41). Experimental metastasis was significantly decreased in Fat-1 mice compared with WT littermates, which was associated with decreased levels of the endothelial marker CD31.

### Effects on Therapeutic Response

Multiple studies suggested addition of fish oil ω-3 fatty acids may enhance the therapeutic response of TNBC to standard chemotherapeutics. Docetaxel is a taxane used as a first line therapy in TNBC (102). In MDA-MB-231 cells, DHA (30 μM) enhanced the cytotoxic efficacy of docetaxel (53). Moreover, treatment with DHA prevented docetaxel-induced increases in membrane localization of PKCε and PKCδ and phosphorylation of ERK1/2 and Akt, which are associated with drug resistance (53). Dietary supplementation with fish oil (5% by weight) was also shown to enhance the therapeutic response of N-methylnitrosourea (NMU)-induced tumors to docetaxel in rats, which was attributed to downregulation of ERK- and Akt-regulated survival pathways. Another study documented addition of DHA to the diet (3.4%) of two different patient-derived TNBC xenograft (PDX) mouse models significantly improved the efficacy of docetaxel (54). Here, authors used the MAXF574 PDX, which is poorly differentiated and well-vascularized and the MAXF401 model, which is moderately differentiated and poorly vascularized. In mice bearing the MAXF574 PDX and treated with docetaxel, dietary DHA resulted in a 64% reduction in tumor weight compared to control-fed mice. Similarly, tumor weight was decreased by 34% in DHA-fed mice bearing the MAXF401 PDX treated with docetaxel compared with control-fed animals. In both PDX models, decreased in tumor volume were attributed to higher expression of the pro-apoptotic factors Ripk1 and Bid; lower expression of the Bcl-2, PARP, and surviving; and induction of cell-cycle arrest.

Doxorubicin is another first-line chemotherapy used in TNBC (103). Studies showed DHA enhanced the efficacy of doxorubicin in TNBC cells and an *in vivo* mouse model (55). Single agent treatment with either doxorubicin or DHA (60 μM) decreased MDA-MB-231 cell viability. Compared to control treatment with only doxorubicin, treatment with DHA and doxorubicin had a stronger effect on cell viability and brought about cell cycle arrest. These changes were attributed to increased expression of several pro-apoptotic factors, including caspase-10 and caspase-9, and decreased levels of cell-cycle progression proteins, including cyclin B1 and WEE1, in DHA-treated cells.

Apatinib is a tyrosine kinase inhibitor that targets vascular endothelial growth factor receptor (VEGFR)2, which has been shown to be upregulated in TNBC patients (104). Addition of apatinib to the treatment regimen of metastatic TNBC patients provided a beneficial outcome in two case reports (105, 106). In MDA-MB-231 cells, single-agent treatment with either apatinib or DHA dose-dependently decreased proliferation (56). On the other hand, treatment with different combinations of apatnib + DHA formulations showed improved efficacy. The optimal dose was found to be 40.5 μM of apatinib (75% of the single-agent IC_50_) in combination with 112.5 μM DHA (75% of single-agent IC_50_). Authors reported these effects were due in part to downregulation of Bcl-2 and phosphorylated Akt and increased levels of cleaved caspase-3 and BAX (56). Together, these studies demonstrate a potential efficacy for adjuvant DHA in TNBC treatment regimens.

## Olive Oil and TNBC: Review

### Hydroxytyrosol and Oleuropein on Proliferation, Migration and Invasion

The effects of olive oil components on TNBC cells are summarized in Table 3. Hydroxytyrosol is a simple phenol found in olive oil at concentrations ranging from 0.71 to 2.7 mg/kg (112). Previous studies had highlighted an anti-tumorigenic potential of hydroxytyrosol due to its capacity to protect against oxidative DNA damage in non-malignant MCF10A breast cells (113). More recently, hydroxytyrosol was shown to have inhibitory effects on breast cancer stem cell (BCSC) self-renewal in a comprehensive investigation using multiple TNBC cell lines (57). Tumor enrichment of BCSC has been suggested to play a role in the aggressiveness and poor prognosis associated with TNBC (114). Hydroxytyrosol at a range of concentrations (0.5–100 μM) suppressed mammosphere-forming efficiency, a marker of BCSC self-renewal, of the TNBC cell lines SUM159PT, BT549, MDA-MB-231, and Hs578T (57). In fact, the 0.5 μM dose was sufficient to suppress mammosphere formation of SUM159PT cells by 40% and the three other TNBC cell lines (BT549, MDA-MB-231, and Hs578T) by 50% after 72 h in culture. Interestingly, treatment with hydroxytyrosol actually increased the size of Hs578T spheres, despite decreasing formation efficiency. Treatment with hydroxytyrosol was also shown to decrease migration of SUM159PT, BT549, and MDA-MB-231 cells, however the effect was less pronounced in SUM159PT cells. In BT549 and MDA-MB-231 cells, these effects were observed in parallel with dose-dependent reductions in phosphorylated LRP6, LRP6, and β-catenin by hydroxytyrosol treatment, which suggested suppression of Wnt/β-catenin signaling. Moreover, hydroxytyrosol treatment was shown to inhibit TGFβ/SMAD2/3-mediated EMT (57).

**Table 3:** Effects of olive oil components on TNBC cells.

**Component**	**Effects**	**Model**	**Target or Mechanism**	**References**
Hydroxytyrosol/Oleuropein	Inhibited BCSC renewal Activated autophagy Decreased Invasion/metastasis Induced apoptosis	SUM159PT, BT549, MDA-MB-231, Hs578T cells MDA-MB-231 cells MDA-MB-231 cells MDA-MB-231 cells	Wnt/β-catenin, TGF-β LC3-II/LC3-I, beclin-1, p62 Autophagy ROS accumulation, NF-κB	(57) (58) (58) (59)
Oleanolic acid	Apoptosis and autophagy Apoptosis and cell cycle arrest Decreased migration	MDA-MB-231 cells MDA-MB-231 cells MDA-MB-231 cells	Akt, NF-κB ROS accumulation Rac1	(60) (62, 107) (65)
Oleic acid	Enhanced proliferation Enhanced invasion Enhanced migration Enhanced chemosensitivity	MDA-MB-231 cells MDA-MB-231 cells MDA-MB-231 cells MDA-MB-231 cells	Akt, PI3K MMP9, NF-κB FFAR1, FFAR4, FAK, SCD1, PLD/mTOR FASN	(64, 108) (64, 109) (64–67, 110) (68)
Oleocanthal	Decreased proliferation, migration, invasion Decreased tumor volume Decreased tumor recurrence Extended tumor latency Decreased proliferation	MDA-MB-231 cells MDA-MB-231 xenografts MDA-MB-231 xenografts MDA-MB-231 xenografts MDA-MB-231 cells	c-MET, TRPC6 c-MET MET, HER2, E-cadherin, vimentin c-MET PI3K/mTOR	(69, 70, 111) (69, 71, 72) (71, 72) (72) (73)
Pinoresinol	Cytotoxic	MDA-MB-231 cells	Pro-oxidant	(74)

Another study demonstrated treatment with hydroxytyrosol and its ester oleuropein (hydroxytyrosol esterified with elenolic acid) reduced MDA-MB-231 cell viability in a dose-dependent manner (58). However, hydroxytyrosol had a more potent effect than oleuropein. Regardless, both compounds inhibited hepatocyte growth factor (HGF)-induced migration and invasion of MDA-MB-231 cells in a manner similar to rapamycin. This was associated with reversal of HGF-induced changes in LC3-II/LC3-I ratio, downregulation of Beclin-1 and upregulation of p62, suggesting these olive oil compounds may suppress migration and invasion of MDA-MB-231 cells by activating autophagy. The IC_50_ of oleuropein against cell viability of MDA-MB-231 and MCF7 luminal A BC cells was 36.2 and 95.4 μM for 72 h, respectively, suggesting a much higher sensitivity of TNBC cells than ER-positive cells (59). Treatment of MDA-MB-231 with oleuropein also decreased migration, induced apoptosis and led to the accumulation of reactive oxygen species (ROS). These changes were linked to the capacity of oleuropein to inhibit nuclear factor-κB (NF-κB) activity.

### Oleanolic Acid on Proliferation and Migration

Oleanolic acid is a triterpenoid abundant in olive oil (26.71–50.13 μg) (75) with previous studies (>5 years prior to this review) that had demonstrated anti-proliferative capacity in both MDA-MB-231 TNBC and MCF7 luminal A BC cells with respective half maximal inhibitory concentrations (IC_50_) of 7.0 and 7.5 μM (107). These anti-proliferative effects were attributed to induced cell cycle arrest and apoptosis in these cell lines. Since then, multiple derivatives of oleanolic acid have been investigated for their anti-BC potential. Methyl 3-hydroxyimino-11-oxoolean-12-en-28-oate, a synthetic derivative, reduced MDA-MB-231 cell viability with an IC_50_ of 21.08 μM by activating apoptotic (extrinsic) and autophagic pathways (60). Another oleanolic acid derivative, SZC017, was shown to inhibit cell viability of both MDA-MB-231 (IC_50_ = 28.09 μM) and MCF7 (IC_50_ = 26.47 μM) (61). Unfortunately, authors used only the MCF7 cell line to investigate the mechanisms underlying these effects, which were attributed to apoptotic and autophagic induction in part by decreasing Akt activation and nuclear accumulation of phosphorylated-p65.

This report is somewhat contrasted by another study which demonstrated a cytotoxic effect of oleanolic acid in MDA-MB-231 cells but not MCF7 cells (62). After 24 h in media containing 100 μM oleanolic acid, only 68% of MDA-MB-231 cells survived, whereas there was no statistical decrease in the number of MCF7 cells. However, these differences could be due to the fact that a much higher dose (100 μM) was used in these experiments. In the ER-negative non-malignant MCF10A cell line, oleanolic acid had a cytotoxic effect at both 10 μM (83% cell survival) and 100 μM (13% cell survival) after 24 h in culture. Oleanolic acid was also shown to inhibit proliferation in a dose-dependent manner after 24, 48, and 72 h in culture and the decrease in proliferation was significant starting at 1 μM. Similar effects were observed in the MCF10A cells starting at the 10 μM dose, however an anti-proliferative effect was only observed in the MCF7 cells at 100 μM. The anti-proliferative effect in MDA-MB-231 cells was ascribed in part to intracellular accumulation of ROS. Interestingly, oleanolic acid also protected against H_2_O_2_-induced DNA damage in MCF10A cells (62).

In addition to anti-proliferative effects, oleanolic acid may also have anti migratory potential in TNBC cells. Treating MDA-MB-231 cells with oleanolic acid suppressed cell migration with an IC_50_ of 14.0 μM (63). Moreover, carbamoylation of oleanoic acid to derive 3-O-[N-(30-chlorobenzenesulfonyl)- carbamoyl]-oleanolic acid (IC_50_ = 2.1 μM) and 3-O-[N-(50-fluorobenzenesulfonyl)-carbamoyl]-oleanolic acid (IC_50_ = 1.4 μM) provided more potent antimigratory effects. Western blot analysis indicated the antimigratory effects of oleanolic acid and its derivatives were linked to inhibition of the breast tumor kinase (BRK)/Paxillin/Ras-related C3 botulinum toxin substrate (Rac1) signaling pathway.

### Oleic Acid on Proliferation, Migration and Therapeutic Response

Oleic acid is the most abundant free fatty acid in olive oil (809.53–1045.52 μg/g) followed by palmitic acid (285.41–362.95 μg/g) (75). Previous studies had documented oleic acid may have a pro-tumorigenic effect on TNBC. For example, in MDA-MB-231 TNBC cells, oleic acid increased proliferation via PI3K activation (108), invasion via (MMP)9 secretion (109) and migration through increased production of arachidonic acid and subsequent activation of focal adhesion kinase (FAK) (110). A recent study demonstrated a 4-fold increase in MDA-MB-231 cell migration by oleic acid (100 μM) was attenuated by concurrent treatment with selective inhibitors for free fatty acid receptor (FFAR)1 and FFAR4, suggesting their involvement in mediating the pro-migratory effect of oleic acid in TNBC cells (64). Selective inhibitors of PI3K and AKT also attenuated oleic acid-induced migration of these cells and subsequent experiments revealed this was due to oleic acid-induced phosphorylation of both AKT1 and AKT2. The increase in migration elicited by oleic acid was demonstrated to require AKT2 expression in MDA-MB-231 cells. Oleic acid also increased the activity of nuclear factor kappa-light-chain-enhancer of activated B cells (NF-μB), a transcription factor that regulates genes involved in invasion, in MDA-MB-231 cells. In summary, this study documented critical roles of FFAR, PI3K, AKT, and NF-κB in mediating the pro-invasion/migration effect of oleic acid on TNBC cells.

Cancer-associated fibroblasts have been shown to promote EMT characteristics and increase migration of MDA-MB-231 and MCF7 BC cells in culture (115). However, this effect is attenuated by knockdown of stearoyl-CoA desaturase (SCD)1, a endoplasmic reticulum desaturase that regulates membrane fluidity via conversion of saturated fatty acids to MUFA (116). Interestingly, treatment of SCD1-knockdown MDA-MB-231 and MCF7 cells with oleic acid restored CAF-mediated pro-migratory effects in wound-healing assays (65). High expression of SCD1 in TNBC was associated with increased proportion of metastasis-related deaths (HR = 6.73) (66). In wound-healing assays, oleic acid treatment increased wound recovery of MDA-MB-231 and luminal A (T47D and MCF7) by 1.5-fold compared with untreated cells (66). However, wound recovery by oleic acid-stimulated MDA-MB-231 cells was attributed to enhanced migration, whereas luminal A cell recovery was due to proliferation. An analysis of migratory dynamics revealed oleic acid increased the speed and changed the directionality of MDA-MB-231 cell migration. These changes were linked to oleic acid-mediated activation of a phospholipase D (PLD)/mTOR signaling pathway. In line with this, treating MDA-MB-231 with rapamycin attenuated oleic acid-induced (250 μM) proliferation, invasion and migration (67). This was accredited to inhibitory effects of oleic acid and activating effects of rapamycin on autophagy in MDA-MB-231 cells.

Cisplatin is a platinum-based chemotherapy that demonstrated efficacy as a first-line chemotherapy in TNBC patients (117). However, multiple mechanisms of cisplatin-resistance have been identified in TNBC cells (118). Despite the potential deleterious effects of oleic acid on TNBC cells, one study demonstrated a potential synergistic relationship between oleate and Cisplatin on inducing apoptosis in MDA-MB-231 cells (68). Treatment with oleate (50 μM) increased the number of cells undergoing apoptosis when used in combination with Cisplatin compared to cells treated with Cisplatin alone, which was attributed to oleic acid-dependent downregulation of fatty acid synthase (FASN). Previous studies had also demonstrated oleic acid enhanced the sensitivity of MDA-MB-231 TNBC cells and T47D and MCF7 luminal A cells to the cytotoxic effects of paclitaxel (119). Although it has not been tested, there may be a potential role for oleic acid in modulating TNBC therapeutic response.

### Oleocanthal on Proliferation, Migration and Invasion

Oleocanthal is a secoiridoid present in olive oil that was previously shown to inhibit proliferation (20 μM), migration (10 μM), and invasion (10 μM) of MDA-MB-231 TNBC cells *in vitro* (111). These effects were attributed to suppression of MET proto-oncogene, receptor tyrosine kinase (c-Met) signaling, a pro-carcinogenic factor upregulated in ~43% of TNBC samples (120). A follow up study demonstrated oleocanthal had a pro-apoptotic effect in these cells and stabilized a more epithelial, rather than mesenchymal, phenotype (69). In mice, (-)-oleocanthal (5 mg/kg) administered via intraperitoneal injection (3X/week) reduced MDA-MB-231 triple-negative xenograft tumor volume by 60% compared to vehicle-treated controls. These *in vivo* outcomes at this dose were attributed to inhibition of c-MET and cytostatic effects rather than a pro-apoptotic influence. Interestingly, the tumor suppressive effects of oleocanthal may be selective to malignant cells. For example, one study showed that (-)-oleocanthal (10–20 μM over 24–72 h) decreased cell viability, proliferation and migration of MDA-MB-231 TNBC cells and MCF7 luminal A BC cells in culture, but had no effect on non-malignant MCF10A cells (70). These differences in effect were linked to downregulation of the Ca^2+^ channel transient receptor potential cation channel subfamily C member 6 (TRPC6), which is overexpressed by the BC cell lines. Previous studies showed silencing TRPC6 decreased proliferation and migration of MDA-MB-231 cells (121).

Another study tested the effect of oral oleocanthal exposure on a xenograft model of breast tumor recurrence (71). These experiments demonstrated the capacity of oral oleocanthal (10 mg/kg daily) to reduce the growth of recurrent MDA-MB-231 xenograft tumors by 58%, which was associated with lower serum levels of cancer antigen (CA)15-3 (71), a tumor marker of post-operative recurrence and metastasis risk (122). Tumors from oleocanthal-treated mice also had decreased phosphorylated c-MET, increased in E-cadherin and downregulation of vimentin (71). A taste-masked oleocanthal formulation using (+)-xylitol as an inert carrier was developed to improve palatability of oral oleocanthal administration (72). Daily oral administration of this formulation (10 mg/kg) to athymic nude mice starting 45 days before orthotopic inoculation of MDA-MB-231 cells decreased mean tumor volume by 49% compared with control mice. This same dose (10 mg/kg) was also shown to decrease MDA-MB-231 tumor volume by 48% when administered to mice 5 days after xenograft inoculation. Mice that received this xylitol-oleocanthal formula after surgical resection of MDA-MB-231 tumors also had extended recurrence-free survival, 60% recurrence prevention, and significantly decreased volume of recurred tumors compared with placebo-treated mice. Again, inhibitory effects on tumor growth were attributed to antagonism toward c-MET.

Mammalian target of rapamycin (mTOR) is a serine/threonine kinase that plays a significant role in cell survival, growth and proliferation. Activity of mTOR is increased via multiple mitogenic signals, namely phosphatidylinositol 3-kinase (PI3K)/AKT serine/threonine kinase (AKT) signaling (123). As is the case for most cancers, aberrations in the PI3K/AKT/mTOR pathway are common in TNBC (124). Molecular modeling studies documented oleocanthal shared with a dual inhibitor of mTOR and PI3K, 9/10 critical binding interactions with mTOR and *in vitro* kinase assays found the IC_50_ for oleocanthal against mTOR activity to be 708 nM (73). In cell culture studies, oleocanthal had strong anti-proliferative effects in MDA-MB-231 TNBC cells (IC_50_ = 18.5 μM) and T47D (IC_50_ = 20.0 μM) and MCF7 (28.0 μM) luminal A cells. Western blotting revealed these effects were linked with decreased phosphorylation (activation) of mTOR in MDA-MB-231 cells treated with oleocanthal.

### Pinoresinol on Proliferation

Pinoresinol is a lignan found in olive oil (0.07–0.9 mg/kg) (125) that is considered a phytoestrogen due to its similar chemical structure to estrogen. Previous studies had demonstrated pinoresinol (100 μM) had anti-proliferative effects on MDA-MB-231 cells (70 ± 6% cell survival) but not MCF7 cells (96 ± 7% cell survival) (126). Recently, pinoresinol treatment was shown to have cytotoxic effects on MDA-MB-231 cells at concentrations ranging from 0.001–100 μM, whereas only low levels (0.001–0.01 μM) were cytotoxic to MCF7 cells (74). Interestingly, the cytotoxic effect of pinoresinol may be specific to tumorigenic cells. This was evidenced by the fact that none of the treatment doses (0.001–0.01 μM) led to >10% cell death in the non-malignant MCF10A cell line. Moreover, the amount of MCF10A cell death with the 0.001 and 0.01 μM pinoresinol treatment was statistically lower than that of MDA-MB-231 and MCF7 cells at those doses. Further investigation revealed an anti-proliferative effect of pinoresinol in the BC cell lines that was associated with a pro-oxidant effect. On the other hand, in the non-tumorigenic cell line pinoresinol had an antioxidant effect, which reduced cellular oxidative stress and H_2_O_2_-induced DNA damage.

## Discussion

Triple negative breast cancers are the most aggressive and lethal of the BC molecular subtypes. Although there have been advances in chemotherapeutic regimens for TNBC (2), there is a potential benefit to identifying lifestyle interventions that target molecular effectors of TNBC initiation and progression. The diet is a key example of a modifiable risk effector that influences a myriad of molecular processes with both positive and negative implications toward mammary tumorigenesis. In both clinical trial (22) and meta-analysis (21, 23, 24), adherence to the MD has been shown to be inversely associated with BC development. Stratified analyses in observational studies identified this relationship to be a more distinct for ER-negative breast cancers in particular (26). Although no meta-analysis has specifically analyzed MD adherence and TNBC incidence, individual observational studies have reported an inverse relationship (27). With this in mind, we sought to review the recent evidence regarding an effect of two principle components of the MD, fish and olive oil, on model systems of TNBC to gain perspective on the role of a MD to aid in TNBC prevention and prognosis.

Components of both fish and olive oil demonstrated antagonistic effects toward several cancer hallmarks in TNBC cells. Both ω-3 essential fatty acids of fish oil, DHA and EPA, had anti-proliferative effects against two different TNBC cell lines, MDA-MB-231 and HCC1806, in cell culture experiments (42). Moreover, in mice, a diet supplemented with EPA and DHA suppressed tumorigenesis of two different mouse-derived TNBC xenograft tumor models (48). Several components of olive oil were also shown to have anti-proliferative effects against TNBC cells including hydroxytyrosol, oleuropein (58), oleanolic acid (107), oleocanthal (111), and pinoresinol (74). Oleocanthal, also inhibited TNBC cell invasion in cell culture experiments (111) and suppressed the formation of primary (69) and recurrent (71) MDA-MB-231 xenograft tumor formation when administered to mice.

Cell proliferation and viability can be decreased through multiple mechanisms including induction of cell death programs (e.g., apoptosis and pyroptosis), suppression of upregulated growth signaling and cell-cycle arrest. Both DHA and EPA were shown to activate apoptosis (42) and DHA activated pyroptosis (43) and modulated cellular energetics (47) in TNBC cells. The anti-proliferative effects of oleuropein (59), oleanolic acid (107), and oleocanthal (69) were also linked to apoptotic induction. On the other hand, hydroxytyrosol and its ester oleuropein were suggested to activate autophagy (58). An attractive aspect of the anti-proliferative effect of several of the components of the MD oils including DHA (43, 44), EPA (44), oleocanthal (70), and pinoresinol (74, 125), is their apparent selectivity toward malignant cells. In these experiments that documented inhibitory effects of these compounds on proliferation of TNBC cells, no effect was observed in the non-malignant MCF10A cell line. It is important to note, the vast majority of studies we reviewed did not include non-malignant control cell lines in their experiments, so one could expect more than just the aforementioned bioactives (DHA, EPA, oleocanthal, pinoresinol) elicit this selective effect.

Activating the EMT and progressing through the metastatic process is a key determinant of BC prognosis as the median survival rate for metastatic TNBC is ~10–13 mo (5). In addition to proliferation, DHA was shown to inhibit migration (44, 49, 51, 52) and invasion (41, 44, 50) of TNBC in culture. Mouse experiments using the tail vein injection model in Fat-1 mice, which produce higher amounts of endogenous ω-3 essential fatty acids, also suggested a potential anti-metastatic effect (41). However, these experiments did not use a human derived TNBC cell line nor an exogenous source of ω-3, which would more resemble exposure to a MD pattern. The olive oil polyphenol hydroxytyrosol also produced an anti-migratory effect in SUM159PT, BT549, and MDA-MB-231 TNBC cells and in this same study decreased BCSC self-renewal in 3D mammospheres (57). Both hydroxytyrosol and its ester oleuropein also inhibited MDA-MB-231 cell invasion (58). An anti-migratory effect was also produced by treating MDA-MB-231 cells with oleanolic acid (63) and oleocanthal (111). Oleocanthal was also anti-invasive against MDA-MB-231 cells (111).

Despite these potential benefits, we would be remiss to not discuss the deleterious effects observed with treatment of TNBC cells with oleic acid, the principle fatty acid in olive oil. The evidence reviewed here suggests oleic acid alone may increase TNBC cell proliferation (108), migration (110), and invasion (109). However, in the context of the MD, oleic acid exposure would occur along with a cocktail of several other dietary components that may abrogate its negative effect. Studies showed inhibitors of PI3K, AKT, and mTOR attenuated oleic acid-induced increases in TNBC cell proliferation, migration, and invasion (64, 67) and the MD oils alone contain several components that antagonize the PI3K/AKT/mTOR signaling pathway. For example, oleocanthal was shown to be an inhibitor of both PI3K and mTOR (73) and both DHA (94) and oleanolic acid (61) inhibited AKT. Thus, there is a strong potential that the pro-tumorigenic effect of oleic acid on TNBC is blunted when delivered as complete olive oil or in the context of a MD.

**Figure 3:**
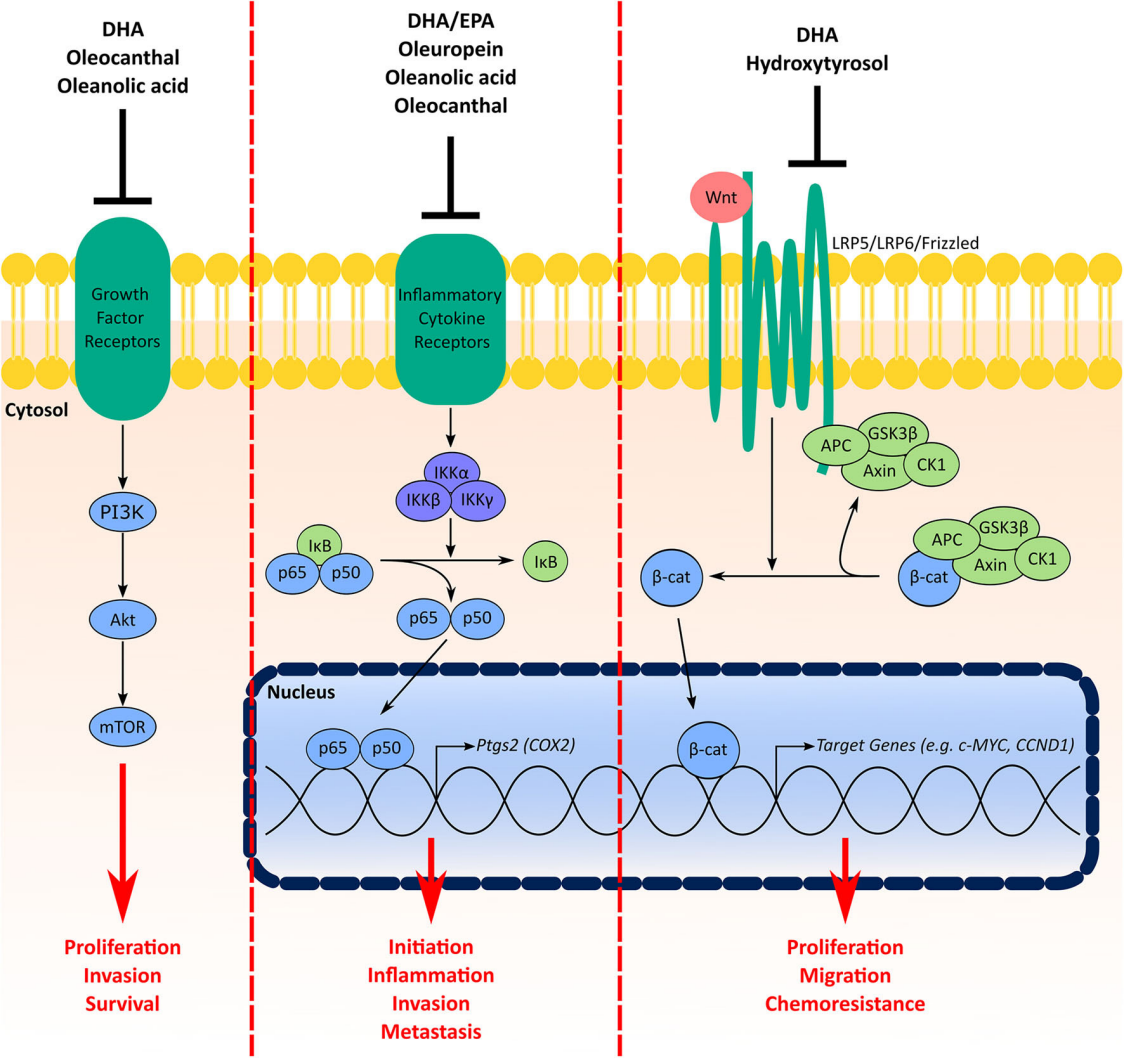
Signaling pathways in TNBC targeted by multiple components of the MD oils. Signaling through the PI3K/Akt/mTOR pathway facilitates cell proliferation, invasion, and survival. Growth factor receptors (e.g., insulin receptor, human epidermal growth factor receptors) activate PI3K, which activates Akt and subsequently mTOR. Components from the MD shown to have inhibitory effects on this pathway include DHA from fish oil in addition to oleocanthal and oleanolic acid from olive oil. COX2 is involved in BC initiation, inflammation, invasion and metastasis. Under basal conditions NF-κB (p65 and p50) is sequestered in the cytosol by inhibitor of κB (IκB) proteins. Activation of inflammatory cytokine receptors (e.g., tumor necrosis factor (TNF) receptor) activates inhibitory κB kinase (IKK) complexes, which phosphorylate IκB proteins for degradation leading to stabilization and nuclear translocation of NF-κB. In the nucleus, NF-κB activates a myriad of target genes, such as *Ptgs2*, the gene encoding the COX2 protein. MD components that inhibit this signaling pathway include the fish oil ω-3 fatty acids in addition to oleuropein, oleanolic acid and oleocanthal from olive oil. Finally, the Wnt/β-catenin signaling pathway increases TNBC cell proliferation, migration and chemoresistance. Under normal conditions, β-catenin is sequestered and degraded in the cytosol by a destruction complex containing axin, adenomatous polyposis coli (APC), glycogen synthase kinase-3 (GSK3)β and casein kinase (CK1). Interaction of Wnt ligands with receptor complexes (e.g., low-density lipoprotein receptor-related protein (LRP)5/LRP6/frizzled) leads to dissociation of β-catenin from the destruction complex, which leads to its stabilization and nuclear accumulation. Both DHA from fish oil and hydroxytyrosol from olive oil have inhibitory effects on the Wnt/β-catenin pathway.

Aberrations in PI3K/AKT/mTOR signaling are common in TNBC and some have suggested this pathway as a therapeutic target (124). Signaling through PI3K/AKT/mTOR influences several cellular processes including proliferation, invasion, survival, metabolism, and chemoresistance and has been shown to play a role in the initiation and progression of mammary tumorigenesis (124, 127, 128). The above example of multiple compounds in the MD inhibiting the PI3K/AKT/mTOR pathway highlights a significant benefit of using a MD-based approach to aid in TNBC prevention and therapy. In addition to PI3K/AKT/mTOR, both NF-κB/COX2 and Wnt/β-catenin are also targeted by components from either fish or olive oil (Figure 3).

Studies have suggested higher expression of COX2 is associated with worse prognosis in TNBC patients (99), BC brain metastasis (98), and tumor inflammation (129). Moreover, COX-2 has been implicated to play a role in BC initiation and invasion (129, 130). Expression of COX2 is induced by NF-κB (131), which is activated by interleukin (IL) receptors, tumor necrosis factor (TNF) receptors, toll like receptors (TLR), and other inflammatory signals (132). In mice bearing CL and BL triple-negative xenografts, a diet enriched with both EPA and DHA decreased tumor expression of COX2 and phospho-p65, the major subunit for NF-kB (48). Activity of NF-κB was also suppressed in tumors of Fat-1 mice bearing EO771 cell xenografts compared wildtype littermates (41). Treatment with DHA also decreased NF-κB activity and COX2 expression in MDA-MB-231 cells (41). The olive oil phenol oleuropein was also shown to decrease NF-κB activity and COX2 levels in MDA-MB-231 cells (59). Oleanolic acid also decreased nuclear accumulation of phosphorylated-p65 (61). Stromal overexpression of COX2 has also been shown to be an independent indicator of worse overall survival in breast cancer (133). Oleocanthal has demonstrated the capacity to downregulate expression of COX2 and activity of NF-kB in adipocytes (134). Given that the breast stroma is largely populated by adipocytes, this collective evidence suggests the MD could facilitate an intra- and extra-tumoral environment that suppresses the NF-κB/COX2 pathway.

Aberrations in the Wnt signaling pathway are common in TNBC [Reviewed here (135)]. Activation of the canonical Wnt signaling pathway stabilizes the transcription factor ω-catenin, which leads to its accumulation and subsequent nuclear translocation (136). ω-catenin controls multiple genes involved in regulating cell proliferation, differentiation/development, and migration (87). Upregulated Wnt/β-catenin confers increased migratory capacity, colony formation, stemness, and chemoresistance of TNBC cells in cell culture and tumor formation of TNBC xenografts in mouse models (137). Interestingly, studies of BC patients have shown nuclear accumulation of ω-catenin in tumors is associated with the TNBC phenotype (138) and upregulated Wnt/β-catenin predicts poor prognosis for TNBC (139). In two studies, DHA had suppressive effects on Wnt/β-catenin-mediated tumorigenicity. Mice fed a diet enriched with EPA+DHA (0.025%) showed a decreased tumor burden from MMTV-Wnt-1 xenografts compared to control-fed littermates (48). In cell culture experiments, DHA dose-dependently decreased expression of ω-catenin in Hs578T TNBC cells and attenuated TPA-induced increases in Wnt-1 levels and secretion and ω-catenin expression in MCF7 cells (51). On the other hand, the olive oil phenol hydroxytyrosol suppressed BCSC renewal and mammosphere formation in BT549 and MDA-MB-231 in part through inhibition of Wnt/β-catenin signaling.

A perplexing question that emerges when examining epidemiological studies investigating the association between MD adherence and BC incidence is: why is there a distinct relationship for ER-negative breast cancers in particular? For example, a meta-analysis suggested the risks for ER- (RR = 0.60), PR- (RR = 0.72), and ER-/PR- (RR = 0.61) BC were significantly decreased with higher adherence to a MD, whereas no associations were found for ER+, PR+, or ER+/PR+ tumors (26). This could be explained in part by the fact that some of the studies we reviewed demonstrated several MD compounds imparted differential effects on proliferation of TNBC cells compared to non-TNBC cells. For example, at 50 μM, DHA produced 85% growth inhibition of MDA-MB-231 TNBC cells compared to only 40% inhibition of T47D luminal breast cancer cell growth (41). Another study actually documented a pro-proliferative effect of DHA in MCF7 luminal cells (45). The olive oil compound oleuropein was shown to have a stronger anti-proliferative effect against MDA-MB-231 cells (IC_50_ = 36.2 μM) compared to MCF7 cells (95.4 μM) (59). Whereas, oleanolic acid treatment was cytotoxic against MDA-MB-231 cells but had no effect on MCF7 cell proliferation (62). The MDA-MB-231 cell line was also more sensitive than MCF7 cells to the cytotoxic effects of pinoresinol (74). Studies have demonstrated both overexpression of COX2 (140) and nuclear accumulation of ω-catenin (138) are associated with the TNBC subtype. Given that MD components covered in this review showed antagonistic effects on both COX2 and ω-catenin, another explanation for an inverse relationship between MD adherence and ER-negative breast cancers could be the specific molecular targets of the MD in TNBC and HER2+ cells. The molecular effects of the MD diet and its constituents on HER2+ breast cancer was outside the scope of this review. However, there is significant evidence to suggest oleic acid and olive oil inhibit HER2+ tumorigenesis and may have therapeutic potential in these cancers [Reviewed here (141)].

Of the studies reviewed here, we identified a general lack of accountability for the heterogeneity of TNBC. As exemplified by Lehmann et al., TNBC can be clustered into several distinct subtypes, which are heterogeneous with respect to clinical parameters such as prognoses and response to therapy (80). The majority of studies we reviewed that performed cell culture experiments used only one TNBC cell line. The most widely used TNBC cell line was the MDA-MB-231 cell line. In addition to the different effects observed between TNBC and non-TNBC treated with MD components, there may be differential outcomes for different TNBC subtypes. We highlighted an example of this with study investigating DHA (45). For MDA-MB-231 cells, 100 μM decreased proliferation by 85% compared to controls, whereas no changes were seen in BT-549 TNBC cell proliferation (45). Moreover, the HCC1806 TNBC cell line was more sensitive to DHA treatment than MDA-MB-231 cells in a different study (42). These differences could be resultant from distinct genetic and molecular features of these cell lines. For example, the MDA-MB-231 cell line has been characterized as mesenchymal stem-like, whereas BT-549 and HCC1806 are considered mesenchymal-like and basal-like 2, respectively (142). BT-549 cells have been shown to harbor a homozygous *PTEN* deletion, whereas both MDA-MB-231 and HCC1806 have been reported to not have aberrations in the PI3K pathway (40). On the other hand, MDA-MB-231 cells are also reported to have mutations in *KRAS*. Another consideration is the fact that the majority of *BRCA1*-mutated tumors (~70%) are TNBC (4), however the MDA-MB-231, BT-549, and HCC1806 cell lines all harbor wildtype *BRCA1* genes (40). This highlights the need for studies looking to impact TNBC prevention and therapy to utilize model systems of TNBC that account for heterogeneity.

The question as to whether or not the MD deserves a role in TNBC prevention and therapy remains unclear at this time. Although we highlighted several examples of fish and olive oil components having anti-tumorigenic effects in TNBC model systems, more work needs to be done in order to optimize clinical recommendations. For starters, MD guidelines call for more specific dietary changes than just increases fish and olive oil consumption. A potentially significant contributor to the effect of MD adherence on breast cancer risk is higher consumption of vegetables and fruits. Unfortunately, a thorough review of the effects of vegetable and fruit bioactive components on TNBC model systems would have exceeded the scope of this current review. However, this is still open for investigation. Furthermore, the potential pro-tumorigenic effect oleic acid may have on TNBC cells demands a careful approach. Although we speculated of a potential entourage effect by delivering oleic acid in the context of a MD, no study has specifically tested if the pro-oncogenic outcomes of oleic acid are abrogated by other MD diet components. This too remains open for investigation.

## Author Contributions

MD, OS, BS, LN, and DR contributed to the conception and development of the manuscript. MD had primary responsibility for the writing of the manuscript. OS and DR contributed to the writing, review, and editing of the manuscript. BS and LN were responsible for the clinical content of the work, contributed to the writing, and review of the manuscript. All authors contributed to the article and approved the submitted version.

## Conflict of Interest

The authors declare that the research was conducted in the absence of any commercial or financial relationships that could be construed as a potential conflict of interest.
